# Development and Application of an IoT-Based System for Soil Water Status Monitoring in a Soil Profile

**DOI:** 10.3390/s24092725

**Published:** 2024-04-25

**Authors:** Alessandro Comegna, Shawcat Basel Mostafa Hassan, Antonio Coppola

**Affiliations:** 1 School of Agricultural Forestry Food and Environmental Sciences (SAFE), University of Basilicata, 85100 Potenza, Italy; shawkat.hassan@unibas.it (S.B.M.H.); antonio.coppola@unibas.it (A.C.); 2 Department of Chemical and Geological Sciences, University of Cagliari, 09042 Cagliari, Italy

**Keywords:** IoT system, capacitive-based sensors, soil water content, soil water retention curve

## Abstract

Soil water content (θ), matric potential (*h*) and hydraulic conductivity (*K*) are key parameters for hydrological and environmental processes. Several sensors have been developed for measuring soil θ–*h*–*K* relationships. The cost of such commercially available sensors may vary over several orders of magnitude. In recent years, some sensors have been designed in the framework of Internet of Things (i.e., IoT) systems to make remote real-time soil data acquisition more straightforward, enabling low-cost field-scale monitoring at high spatio-temporal scales. In this paper, we introduce a new multi-parameter sensor designed for the simultaneous estimation of θ and *h* at different soil depths and, due to the sensor’s specific layout, the soil hydraulic conductivity function via the instantaneous profile method (IPM). Our findings indicate that a second-order polynomial function is the most suitable model (*R*^2^ = 0.99) for capturing the behavior of the capacitive-based sensor in estimating θ in the examined soil, which has a silty-loam texture. The effectiveness of low-cost capacitive sensors, coupled with the IPM method, was confirmed as a viable alternative to time domain reflectometry (TDR) probes. Notably, the layout of the sensor makes the IPM method less labor-intensive to implement. The proposed monitoring system consistently demonstrated robust performance throughout extended periods of data acquisition and is highly suitable for ongoing monitoring of soil water status.

## 1. Introduction

The soil unsaturated zone (or the vadose zone) is the connection zone between the atmosphere and the groundwater and, from a hydrological point of view, plays a fundamental role in the partition of precipitation between surface water, soil water and groundwater [1,2,3,4]. The amount of water that enters the soil is stored in the vadose zone and, from here, can be subjected to several hydrological processes that cause water to move within the soil profile. Estimation of water availability and water flux in the vadose zone provides fundamental information on the soil water balance, which depends on climatic variables and on soil physical and hydraulic properties [5,6]. The availability of such information can have great importance in tackling hydrological, environmental and agricultural issues [7,8,9]. 

In irrigated areas, for example, in-depth knowledge of the soil hydraulic parameters is crucial for crop growth and productivity, as well as for limiting leaching of water, nutrients and other substances (e.g., pesticides) through the unsaturated zone [10,11,12,13,14,15]. In this context, in recent decades, researchers have developed several techniques to estimate soil hydraulic properties, both in field and laboratory conditions. All of the above techniques require sensors for measuring either the water content or the pressure head or both.

With reference to soil water content measuring systems, most determine water content based on the change in some thermal or electrical properties of the soil [16,17,18,19]. As for sensors based on the measurement of electrical quantities, their operation can be based on the propagation of electromagnetic (EM) waves in a medium, which is called dielectric. Belonging to the latter category of sensors are those based on time domain reflectometry (TDR) and frequency domain reflectometry (FDR) techniques, as well as capacitive-based sensors [20,21]. The latter are known to work at low frequencies, and measurements are affected by soil texture, temperature and salinity [22,23,24,25]. Extensive research has demonstrated that such sensors are not so prone to temperature and salinity factors if working in the soil temperature range 15–30 °C and for soils where soil solution electrical conductivity EC_w_ is less than 10 dS/m [26]. However, as measurement frequency increases (around 50 MHz and above), this influence is reduced [27] (Meter Group, online technical note on soil moisture sensors).

Furthermore, capacitive sensors also exhibit varying responses when installed in sandy versus clayey soils due to the distinct porosity and water retention properties of the respective soils. In sandy soils, with their larger particle sizes and higher porosity, water tends to drain more rapidly, resulting in faster sensor response times but potentially lower accuracy in capturing subtle moisture changes. Conversely, in clayey soils with finer particles and greater water retention capacity, water movement is slower, leading to delayed sensor responses and possibly more stable readings over time. Logsdon and Laird [28] also highlighted that clay minerals demonstrate ongoing dispersion beyond frequencies of 100 MHz. This phenomenon significantly affects the determination of water content in clayey soils, as demonstrated by Robinson et al. [29]. To mitigate this issue, manufacturers typically develop factory calibrations under controlled laboratory conditions using soil media with known physico-chemical and dielectric properties [30].

Soil matric potential is the second Kernel soil hydraulic property. It is an integral over the macroscopic interfacial tension of the pore scale menisci of all air–water–soil interfaces. Tensiometers have been used to measure this hydrological variable [31]. However, for large-scale applications, extensive soil hydrologic characterizations are generally required due to their spatial and temporal variability [32], which may require monitoring water content and pressure head in several sites simultaneously. This commonly implies high monitoring costs, especially when sensors have to be connected in a network and the monitoring system includes automatic monitoring and telemetry, with data transmitted in real time using mobile communication technologies [6]. This aspect may contribute to limiting adoption of such sensing technologies [23,33].

To overcome such problems, several low-cost monitoring systems have been developed for soil hydraulic characterization [34]. Most of these devices are noninvasive, with a fast response, no radiation, flexibility in electrode design and, above all, are amenable for automation in the framework of IoT systems [29,35,36,37,38,39,40]. 

In this context, the aim of this research was to design and implement a compact IoT-based platform for stand-alone soil water status monitoring, using several sensors which can be remotely controlled. Our IoT platform allows measurements of volumetric water content (θ) and soil matric potential (*h*) at different depths. Furthermore, the specific layout of the measuring system also allows the K(θ) or K(*h*) relationship to be estimated via the instantaneous profile method (IPM; [41]).

## 2. Materials and Methods

### 2.1. Conceptual Framework of the Monitoring System

The basic apparatus of the monitoring system (named Soil HYdraulic PROperties Meter: SHYPROM) consists of (i) a microcontroller board, (ii) three pairs of steel electrodes for soil moisture estimates, (iii) a pair of porous tensiometer cups connected to two electronic pressure transducers for soil water matric potential measurement, and (iv) a temperature sensor.

**Figure 1:**
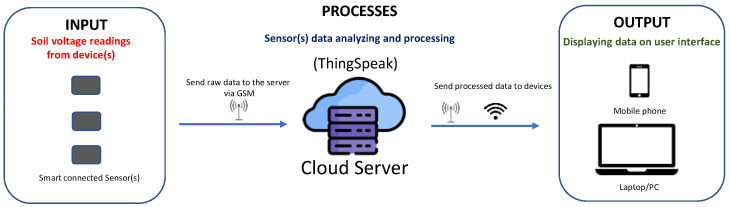
Conceptual framework of the IoT-based sensor platform.

Raw data collected from sensors are managed by the microcontroller and wirelessly transferred to a Cloud Server (e.g., ThingSpeak, https://thingspeak.com/) at fixed time intervals. After sending on Cloud, stored data can then be displayed on an end device (e.g., laptop, mobile phone, etc.). To facilitate sensor data visualization on mobile devices, a customized Android-based application (named SHYPROM_APP) was also developed. Figure 1 shows the schematic diagram of the IoT-based device platform.

### 2.2. Development of the IoT-Based Monitoring System

Nowadays, there is a wide range of commercially available microcontrollers. In the present project, we selected the ESP 32 SIM800L as a microcontroller mainly because of its multiple 12 bits Analogical-to-Digital Converter (ADC) inputs and the capability to generate Pulse Width Modulation (PWM) signals at higher frequencies than other microprocessors such as Arduino Uno. Furthermore, the microcontroller features an integrated nano SIM card slot, as well as WIFI and Bluetooth modules, facilitating remote sensor control.

The device comprises two plexiglass tubes (2 cm in diameter) serving as tensiometers. Each tensiometer is assembled with a porous ceramic cup with an air entry value equal to 150 kPa and an electronic pressure transducer (MPX5100DP model, Freescale Semiconductor, Austin, TX, USA). The pressure transducer includes integrated signal amplification and temperature compensation circuits, supporting a linear voltage output for an overall differential pressure range from 0 to 100 kPa. Each pressure transducer was glued onto a three-way valve, allowing the opening and closing of the tensiometer for water filling. The valve was installed onto a silicone stopper, closing the top side of the tensiometer.

**Figure 2:**
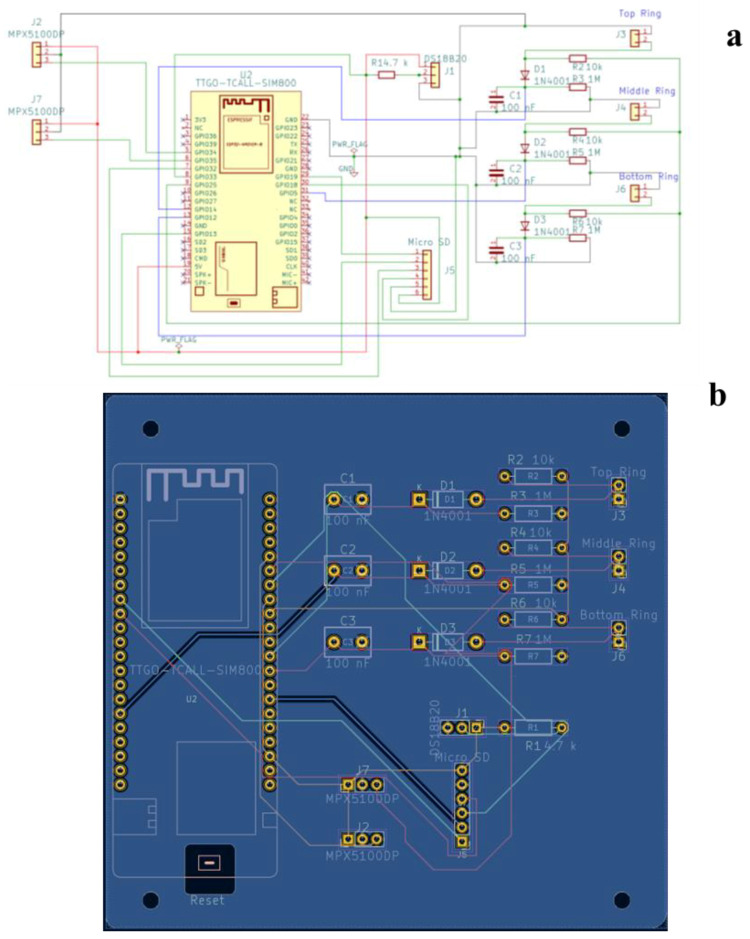
(**a**) Electric circuit diagram of the prototype and (**b**) printed circuit board (PCB) generated by Kicad software (version 7.0).

The soil moisture sensing device is a self-made capacitance-based apparatus, consisting of three pairs of electrodes that operate as a capacitor. The electrodes have a tubular shape and are made of stainless steel 304 (a chromium (18%)–nickel (8%) austenitic alloy). These cylinders (3 cm high and 2 mm thin) were positioned at different heights on the two plexiglass tensiometer tubes, forming three pairs of electrodes. To prevent sensor deterioration, after installation, the electrodes were isolated by spraying with a water-based paint. Additionally, the part of the tube between two electrodes was covered with PLA cylinders (obtained via 3D printing) to protect the wires soldered to the electrodes. The center of the electrodes was located at 11.5 cm, 18.5 cm and 25.5 cm from the top of the plexiglass tubes, the distance between the center of the two tensiometers being set at 8.0 cm. The microcontroller has an integrated oscillator operating at 600 kHz, driving the three capacitors and generating outputs (i.e., the soil’s voltage) proportional to the soil’s dielectric constant (ε, see Appendix A for more details). Figure 2a,b illustrate the wiring diagram and the printed circuit board (PCB) for the proposed monitoring system.

**Figure 3:**
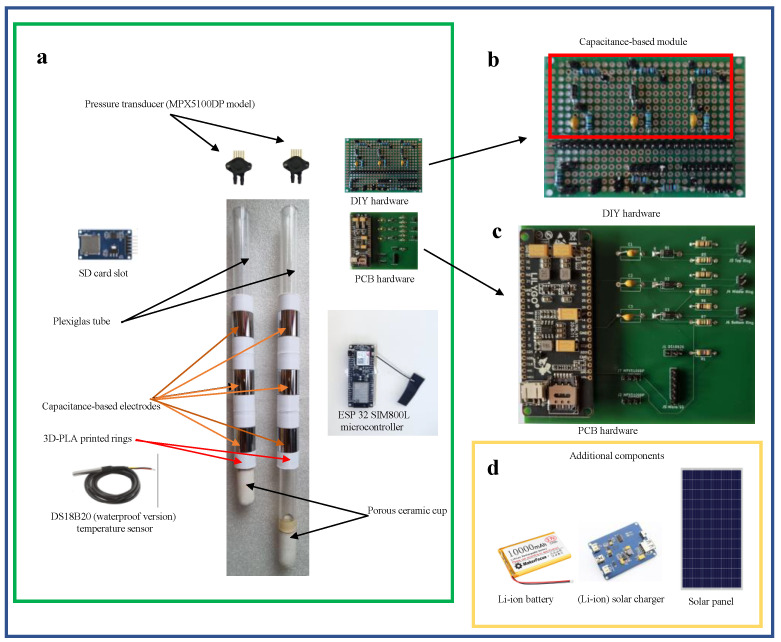
Multiparameter sensor hardware: (**a**) main components, (**b**) detail of the DIY board, (**c**) details of the printed circuit board (PCB), and (**d**) additional material.

Since capacitance measurements are influenced by soil temperature, the device includes a temperature sensor. Specifically, the DS18B20 (waterproof version, Dallas Semiconductor, Dallas, TX, USA) sensor was selected. The sensor operates in the temperature range of −55 °C to +125 °C (with ±0.5 °C accuracy from −10 °C to +85 °C). The device is also equipped with an external micro SD card slot to store raw data in the event of no connection with the Cloud Server. Figure 3a–d display the hardware components of the sensor. The prototype is powered by a Li-ion battery (3.7 V, 10,000 mAh), charged through a solar charger board connected to a waterproof 6 W solar panel. The printed circuit board (PCB) of Figure 3c was designed in Kicad software (https://www.kicad.org/), resulting in a board size of 95 mm × 100 mm. Table 1 presents a list of electronic components (also known as BOM: Bill Of Materials) required for assembling the IoT-based monitoring system (detailed information on the assembly of the monitoring system is reported in Appendix B).

### 2.3. The Source Code Used to Program the Monitoring System

**Figure 4:**
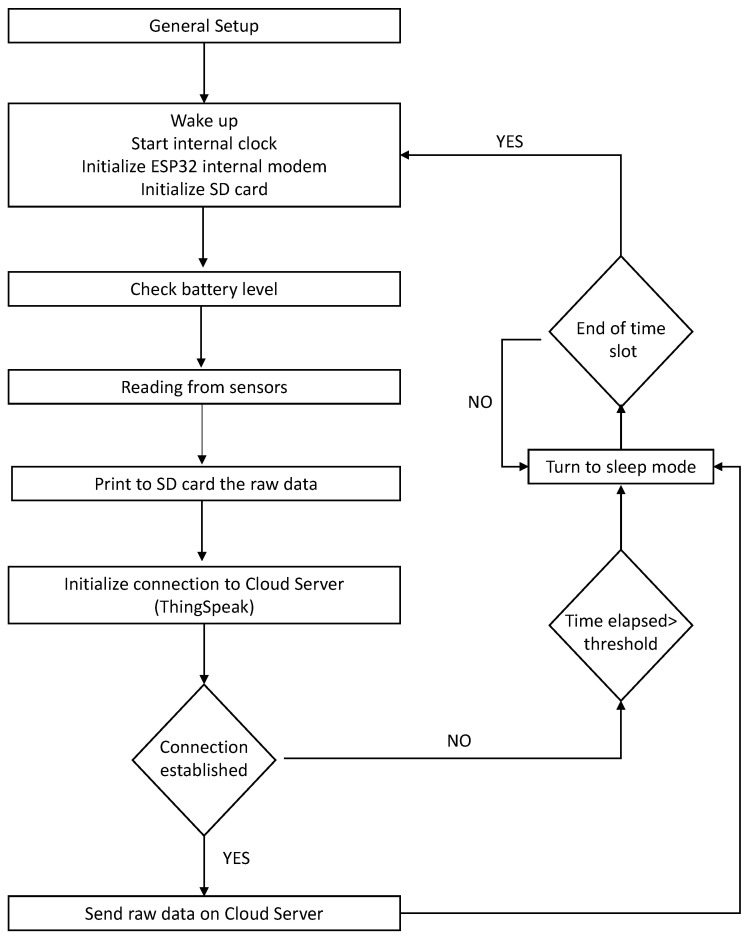
Flowchart of the IoT-based monitoring system.

The source code which governs the hardware of the proposed monitoring system was written in C^++^ using the Arduino Integrated Development Environment (IDE). The architecture of this monitoring system is illustrated in the flowchart of Figure 4. Initially, the microcontroller initiates the general setup, which involves exiting sleep mode, initializing the SD card module and activating the internal modem. At this stage, the algorithm conducts a battery check before commencing the readings from various sensors: (i) soil temperature, (ii) soil voltage from the capacitive module at three distinct depths, and (iii) voltage output from tensiometers at two selected depths. Once obtained, the data are stored on the SD card and transmitted to the Cloud Server. Subsequently, the sensor enters sleep mode to minimize power consumption until the designated time slot concludes.

### 2.4. Evaluating the Soil K(θ) Function through the Instantaneous Profile Method (IPM) Using SHYPROM

The IPM (also known as the internal drainage method) [41] is a widely recognized technique for in situ determination of the *K*(θ) function of subsurface layers during unsteady or transient processes [1]. This method relies on simultaneous measurements over time of volumetric water content and the soil water matric potentials at different depths along the soil profile. The procedure involves the installation of various probes, such as time domain reflectometry (TDR) or capacitive probes and tensiometers, to measure the hydraulic properties of the investigated medium. The soil is wetted to achieve saturation, and then the *K*(θ) curves are determined during either a drainage or evaporation process [42,43,44,45]. The obtained information is crucial in addressing environmental and agricultural challenges, particularly for estimating water drainage below the root zone and determining the soil water balance [46].

The IPM is based on the assumption that the amount of water stored, *W*, in a given soil profile and the water flux density, *q*, at any depth *z* and any time *t*, can be calculated as [1]:

(1) $$W\left(z,t\right)=\int_{0}^{z}\theta \left(z,t\right)dz$$

(2) $${q\left(z,t\right)}_{z}=\int_{0}^{z}\frac{\partial \theta \left(z,t\right)}{\partial t}dz={\left.\frac{\partial W\left(z,t\right)}{\partial t}\right|}_{z}$$

Darcy’s law for water flow, at any depth and any time, can be written as [45]:

(3) $${q\left(z,t\right)}_{z}={K\left(\theta \right)}_{z}{\left.\frac{\partial H\left(z,t\right)}{\partial z}\right|}_{z}$$

where *H* is the hydraulic head.

Substituting Equation (2) into Equation (3) and solving for ${K\left(\theta \right)}_{z}$ produces, at the reference plane *z* = *z*_4_ (i.e., the reference depth at which *K* has to be calculated):

(4) $$K\left(\theta \right)=\frac{{\left.\frac{\partial W\left(z,t\right)}{\partial t}\right|}_{z=z_{4}}}{{\left.\frac{\partial H\left(z,t\right)}{\partial z}\right|}_{z=z_{4}}}$$

**Figure 5:**
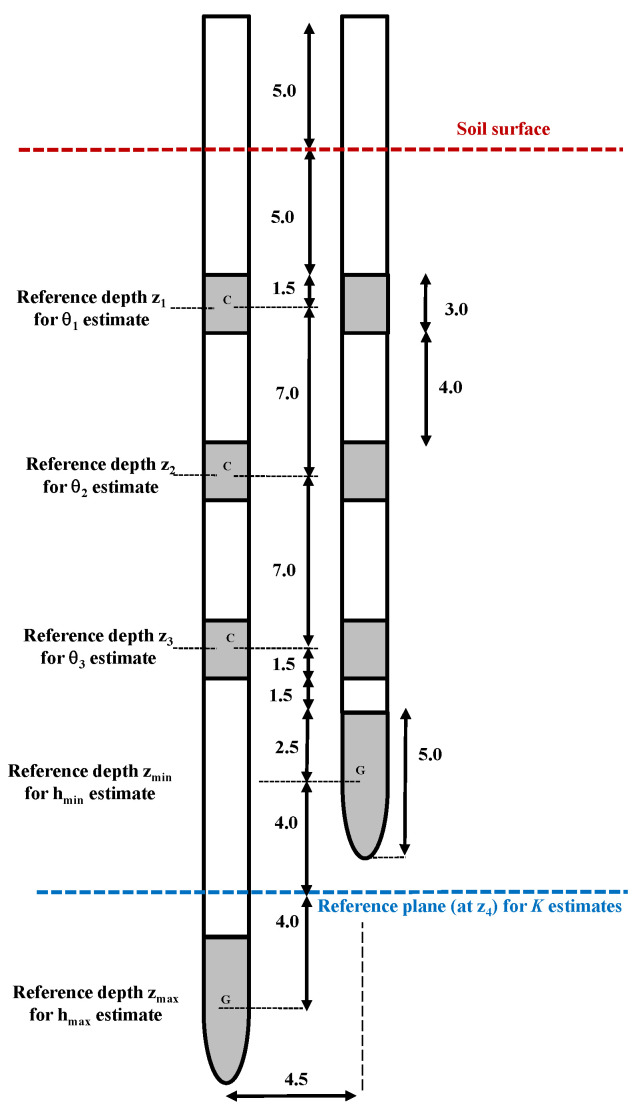
Schematic layout of the SHYPROM monitoring system employed for the laboratory experiments.

The soil conductivity function, at the reference plane, can be estimated under the following hypothesis [47]: (i) the volumetric water content at soil surface (θ_0_) was assumed to be equal to θ_1_; (ii) at the reference plane (z_4_), the volumetric water content (θ_4_) was assumed to be equal to θ_3_. Figure 5 illustrates the schematic layout of the SHYPROM system used in our laboratory experiments.

### 2.5. Soil Characterization

The soil used in our laboratory experiments was a silty-loam Anthrosol [48], with 15.7% sand, 11.6% clay and 72.7% silt. The soil had a bulk density (*ρ_b_*) of 1.13 g/cm^3^, organic *C* content of 1.84%, electrical conductivity of the soil solution (*EC_w_*) of 0.17 dS m^−1^ and a *pH* of 8.40. Soil texture, soil bulk density, organic content and *pH* were determined using the methods proposed by [49,50,51,52]. *EC_w_* was obtained via a conductivity meter (Cyberscan model 500, Eutech Instruments, Singapore).

### 2.6. Laboratory Experiments

Two main sets of experiments, labelled as experiment #1 and experiment #2, were conducted. Experiment #1 focused on the calibration and validation of the sensors. The capacitive sensor underwent calibration and validation during an evaporation process from a bare soil sample. Regarding the MPX5100DP transducers, which measure soil water matric potential (*h*), these sensors come with their own factory calibration function. In this case, we only verified that the pressure transducers operated according to the calibration function provided by the manufacturer.

In experiment #2, the system was tested to measure θ and *h* at different depths, following again an evaporation process. During this stage, the IPM was used to obtain the *K*(θ) relationship.

**Figure 6:**
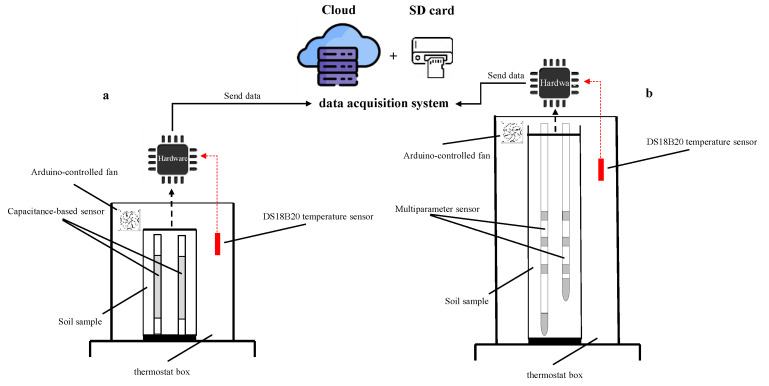
Experimental setup used in (**a**) experiment#1 and (**b**) experiment#2.

For both experiments, the soil samples were preliminarily oven dried at 105 °C and then sieved at 2 mm. Figure 6 shows a schematic representation of the measurement system used in the laboratory experiments.

#### 2.6.1. Experiment #1: Sensor Calibration and Validation

In this experiment, measurements were carried out on a repacked soil sample 90 mm in length and 70 mm in diameter using a capacitive sensor made of a single pair of electrodes. The bottom end of the soil sample was held with a nylon gauze (25 μm) to avoid soil losses. After packing, the electrodes of the capacitive sensor were inserted vertically into the soil column. At the beginning of the test, the soil sample was saturated with water from the bottom to prevent air bubbles being trapped in soil pores. For the calibration procedure, once saturated, the soil sample was placed into a thermostat box and water was allowed to evaporate from the top of the soil sample. Throughout the evaporation test, differences in voltage *V* and θ values (obtained via the thermo-gravimetric method; [53]) were monitored over time. The collected data were plotted on a Cartesian plane θ vs. 1/*V* and subsequently utilized to derive the sensor calibration function.

As stated above, capacitive sensors are influenced by soil temperature. Thus, tests were conducted at three different temperatures of 20 °C, 25 °C and 30 °C. For each selected temperature, three replicates were performed. Additionally, an independent dataset was prepared, using the same experimental protocol, for sensor validation.

#### 2.6.2. Experiment #2: Laboratory Testing of the SHYPROM Monitoring System

Once the sensors had been calibrated and validated, the monitoring system was tested overall, using the same experimental setup and protocol as in experiment #1. In this final test, the sensor probes were placed in a soil sample 400 mm high and 250 mm in diameter. Three evaporation tests were conducted at the temperature of 25 °C, during which measurements of θ and *h* were carried out.

### 2.7. Statistical Indices for Sensor Performance Evaluation

The performance of the calibration function was quantified by using different statistical indices: (i) the mean bias error (*MBE*), (ii) the mean absolute percentage error (*MAE*) and (iii) the model efficiency (*EF*), computed according to the following relations [54,55]:

(5) $$MBE=\frac{\sum_{i=1}^{N}\left(E_{i}-O_{i}\right)}{N}$$

(6) $$MAE\left(\%\right)=\frac{\left|E_{i}-O_{i}\right|}{N}\cdot 100$$

(7) $$EF=1-\frac{\sum_{i=1}^{N}{\left(E_{i}-O_{i}\right)}^{2}}{\sum_{i=1}^{N}{\left(O_{i}-\bar{O}\right)}^{2}}$$

where $E_{i}$ is the prediction (model-simulated data) and $O_{i}$ is the true value (observed data), $\bar{O}$ is the mean of the observed data, and *N* is the number of observations.

## 3. Results and Discussions

### 3.1. Calibration and Validation of the Capacitive Sensor

**Figure 7:**
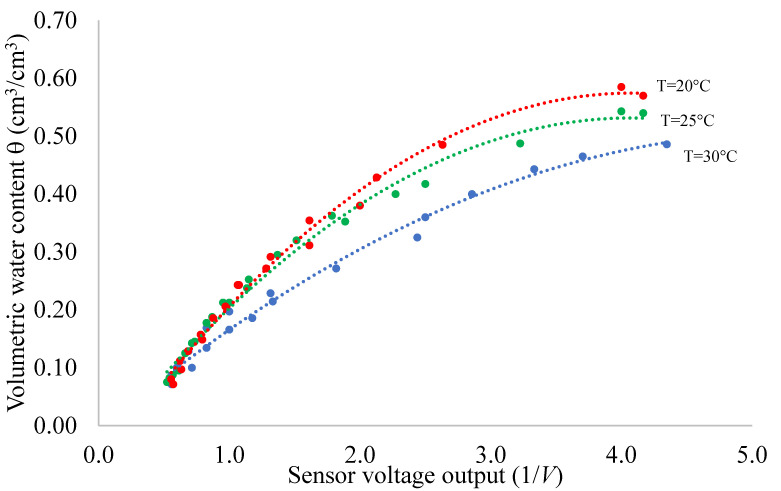
Relationship between volumetric water content θ and the inverse of voltage readings (1/V) for the soil investigated at the three selected soil temperatures T of 20 °C, 25 °C and 30 °C.

The accuracy of capacitive-based sensors is influenced, amongst other things, by temperature fluctuations. The outcomes of experiment #1, illustrated in Figure 7, show this dependence with reference to the proposed monitoring system. The measured θ values are plotted against 1/*V* for the three chosen temperatures. For a fixed temperature, the dashed lines represent the regression functions selected to estimate the correlation between the sensor output and volumetric water content. It is worth noting that, for a fixed sensor voltage value, as soil temperature increases, the θ values decrease. Observable differences in θ values become less evident as the soil becomes dry, since the dielectric response of the sensor is more dependent on soil permittivity.

A second-order polynomial equation was employed to fit the experimental values, and the computed coefficients (*a*, *b* and *c*) along with the coefficient of determination *R*^2^ are detailed in Table 2. Notably, the volumetric water content and sensor output exhibit a strong correlation, as evident from the *R*^2^ values approaching unity.

**Figure 8:**
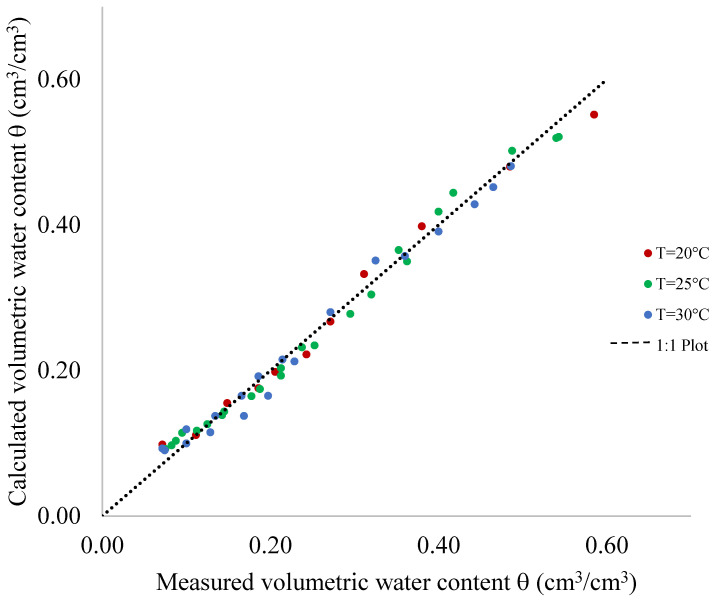
Correlation between the observed and measured values of volumetric water content θ at the temperatures T of 20 °C, 25 °C and 30 °C.

In Figure 8, the θ values derived from regression functions are compared with the known θ values from an independently acquired dataset dedicated to model validation. The obtained values closely align with the 1:1 line, indicating the capacitive sensor’s accurate performance in θ estimation from saturation to dry soil conditions. The sensor’s accuracy is practically the same from saturation to dry soil conditions and among different temperatures.

For comprehensive evaluation, Table 3 summarizes statistical indices, including *MBE*, *MAE* and *EF*, which assess the goodness of fit between calculated and measured θ values. Across different soil temperatures, these indices affirm the sensor’s effectiveness in predicting volumetric water content within the temperature range of 20–30 °C.

*MBE* and *MAE* values, respectively, vary between −0.00014 (at 20 °C) and −0.00030 (at 30 °C) and between 1.14% (at 25 °C) and 1.69% (at 30 °C). The EF exhibits a consistently high value, falling within the range of 0.85 to 0.89.

### 3.2. SHYPROM Laboratory Testing

**Figure 9:**
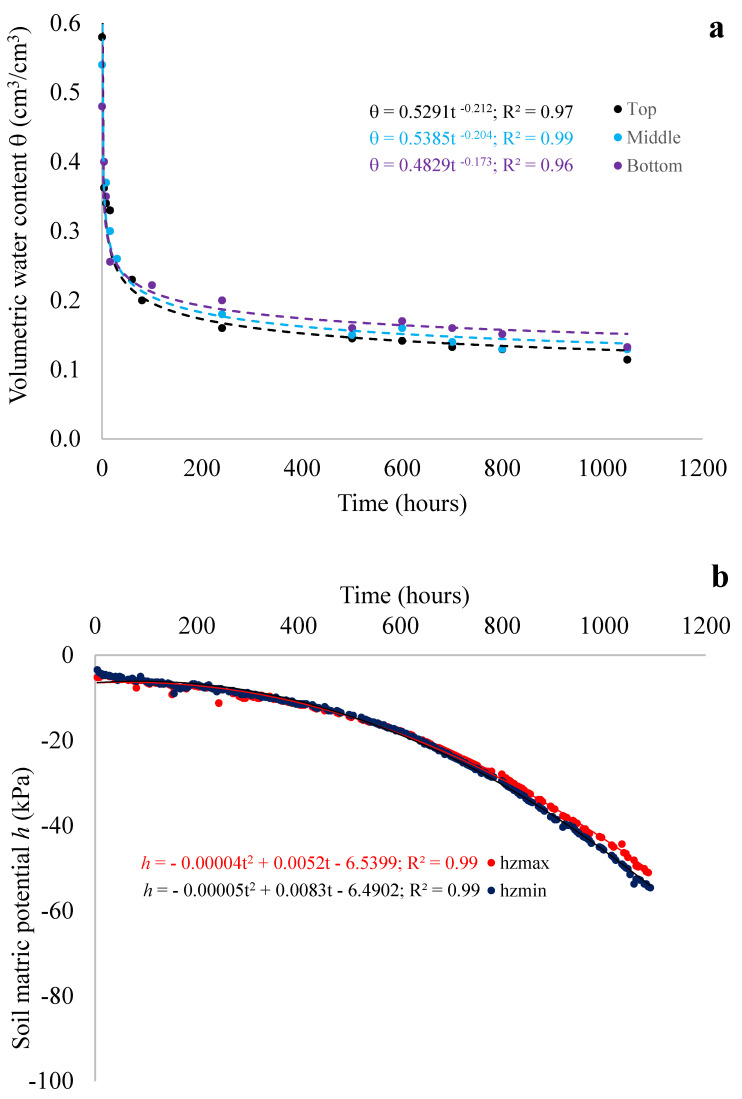
Experimental (data points) and calculated (lines) (**a**) volumetric water content θ at three depths, z_1_, z_2_ and z_3_, and (**b**) matric potential *h* at depths z_min_ and z_max_ vs. time.

An overview of the outcome of the SHYPROM monitoring system is provided in Figure 9a,b. Figure 9a illustrates the relationships between volumetric water content and time at different depths. The graphic also includes polynomial functions used to fit the raw data and the coefficient of determination *R^2^* for the regression functions, which range between 0.96 and 0.99. During the initial stages of the evaporation process, which last around 60 h, the regression functions overlap. As expected, as the process progresses to higher times, the moisture content decreases from the bottom to the top of the soil sample.

Figure 9b displays the measured soil matric potentials as a function of time. Experimental *h* data were fitted, at the selected depths z_min_ and z_max_, using a second-order polynomial function with *R*^2^ equal to 0.99 for both investigated depths.

It is evident that, at a given time, the variations in h values are minimal. This can be attributed to the relatively small distance between the tensiometers.

**Figure 10:**
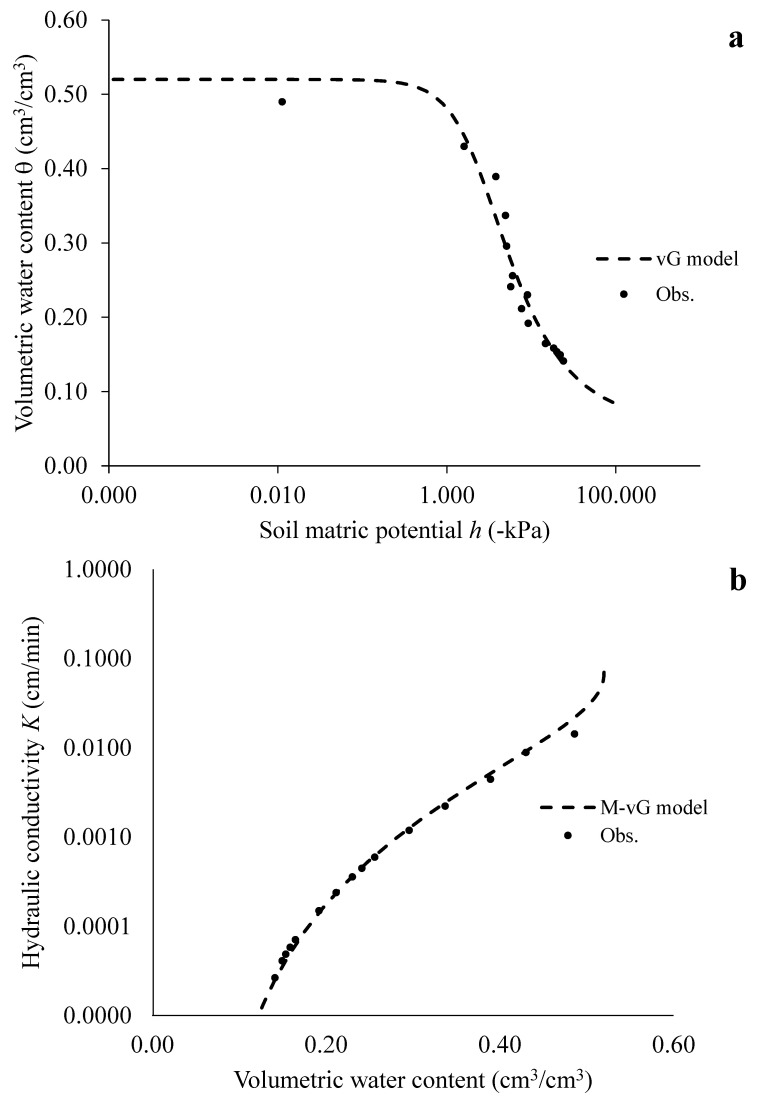
(**a**) Experimental soil retention curve and modelled by van Genuchten (vG) equation and (**b**) experimental soil hydraulic conductivity function and modelled by Mualem–van Genuchten (M-vG) formula at the reference plane z_4_.

Figure 10a,b illustrate the θ(*h*) and *K*(θ) relationship at the reference depth z_4_; *K* values were determined via the IPM method using Equation (4). The empirical retention model by van Genuchten [56] and van Genuchten integrated with the Mualem [57] expression were used to predict, respectively, the experimental θ(*h*) and *K*(θ) functions.

The unknown parameters θ_s_, θ_r_, *α*, *n, m* and *K*_0_, listed in Table 4, were determined by means of the RETC optimization software package, version 6.0 [58], based on the least square method, which minimizes the deviations between the numerical solution of the one-dimensional transient evaporation process and the real response of the system during the laboratory tests. The high *R^2^_vG_* and *R^2^_M-vG_* values denoted that raw data estimated via the SHYPROM monitoring system are of good quality.

The results presented in this section demonstrate that the proposed monitoring system, with its specific layout, accurately characterizes the temporal evolution of the water status in the selected soil.

## 4. Conclusions

Capacitive sensors have been demonstrated to offer precise monitoring of soil water status, serving both environmental and agronomic purposes. With a soil-specific calibration, this category of sensors can serve as a viable, low-cost alternative to the time domain reflectometry technique, which is widely employed for soil water content estimation.

The accuracy of capacitive-based sensors is influenced by various factors such as soil type, moisture levels, soil conductivity or salinity, and temperature fluctuations. These factors warrant further exploration, particularly under field-scale conditions, especially in arid and semi-arid regions with diverse irrigated soil landscapes. In such contexts, the deployment of soil water sensors becomes imperative for optimizing the utilization of limited water resources.

In this study, a low-cost multi-parameter sensor, designed in the framework of IoT systems, was developed and calibrated for measuring water status in a soil sample. The use and applicability of the sensors (a capacitive-based probe to estimate θ and an electronic tensiometer for *h* measurements) were investigated on a silty-loam soil.

Laboratory-scale tests were conducted to assess sensor performance. Specifically, for the capacitance-based module, calibration functions were established at different temperatures to ensure accurate θ measurements. The sensors showed a polynomial behavior, in the investigated domain, with acceptable validation statistics. The specific layout of the sensors allowed estimation of the *K*(θ) function via IPM.

Our results indicate that the monitoring system demonstrated acceptable accuracy from saturated to dry soil conditions. To properly evaluate the potential of the proposed monitoring system, future research will aim to investigate sensor response in soils with different textures and pedological characteristics, as well as the effects of soil salinity on the dielectric response of the capacitive module. Finally, in the upcoming phases, comprehensive field-scale tests will be carried out to evaluate sensor performance under real field conditions.

## Figures and Tables

**Table 1:** List of main electronic components required for making the monitoring system.

Sensor Unit	Designator	Quantity	Material Type
Microcontroller board	ESP32SIM800L	1	Semiconductor
Tensiometer	MPX5100DP	2	Semiconductor
Capacitive Soil Moisture	Resistor 10 kΩ	3	Other
	Resistor 1 MΩ	3	Other
	1N4007 (High Current Rated Diode)	3	Semiconductor
	Capacitor 100 nF	3	Ceramic
Temperature	DS18B20	1	Other
	(pullup) resistor 4.7 kΩ	1	Other
Additional components	micro SD card slot	1	Other
	Li-ion battery (3.7 V, 10,000 mAh)	1	Other
	solar charger board	1	Other
	Solar panel	1	Other

**Table 2:** Estimated regression coefficients a and b and coefficient of determination *R*^2^ of the θ vs. 1/*V* experimental relationships for the three selected soil temperatures.

Soil Temperature (°C)	*a*	*b*	*c*	*R* ^2^
20	−0.0390	0.317	−0.073	0.99
25	−0.0348	0.2834	−0.0459	0.99
30	−0.0181	0.1933	−0.0097	0.99

**Table 3:** Statistical indices mean bias error (*MBE*), mean absolute percentage error (*MAE*) and model efficiency (*EF*) referring to the measured and predicted volumetric water content θ for the three selected soil temperatures.

Soil Temperature (°C)	*MBE*	*MAE (%)*	*EF*
20	−0.00014	1.17	0.89
25	−0.00021	1.14	0.87
30	−0.00030	1.69	0.85

**Table 4:** van Genuchten (vG) and Mualem–van Genuchten (M-vG) model parameters θ_s_, θ_r_ *α*, *n*, *m*, *K_0_* and coefficient of determination *R*^2^*_vG_* and *R*^2^*_M-vG_* obtained from experimental retention and conductivity functions with reference to the selected soil.

θ_s_ (cm^3^/cm^3^)	θ_r_ (cm^3^/cm^3^)	*α* (1/cm)	*n*	*m =* 1 *−* 1/*n*	*K*_0_ (cm/min)	*R* ^2^ *_vG_*	*R* ^2^ *_M-vG_*
0.52	0.055	0.048	1.743	0.426	0.070	0.97	0.96

**Figure A1:**
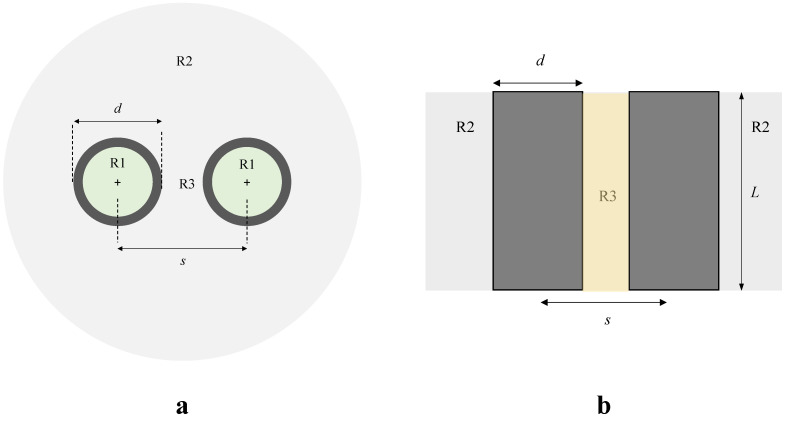
Regions of influence for a capacitor with a parallel wire geometry: (**a**) top view; (**b**) frontal view.

## Data Availability

The original contributions presented in the study are included in the article/supplementary material, further inquiries can be directed to the corresponding author.

## Supplementary Materials

The following supporting information can be downloaded at: https://www.mdpi.com/article/10.3390/s24092725/s1.

## Appendix A. Theoretical and Operational Principles of the Capacitive-Based Module for Volumetric Water Content Estimate

Capacitance sensors work as a capacitor. A capacitor consists of two conductors (or electrodes) separated by an electrical insulator material known as a dielectric. Most capacitance sensors are based on parallel plate theory to measure soil moisture [24,59]. In such sensors, capacitance (*C*) describes how two conducting objects (i.e., the conductors) with a space between respond to the voltage *V* applied between them:

(A1) $$C=\frac{Q}{V}$$

where *Q* is the (positive or negative) electrical charge stored on the capacitor. Equation (A1) holds true for an ideal capacitor (i.e., characterized by a constant capacitance *C*). In practical devices, charge build-up sometimes affects the capacitor mechanically, causing its capacitance to vary. In this case, capacitance can be defined in terms of incremental changes:

(A2) $$C=\frac{dQ}{dV}$$

Capacitance is significantly influenced by the dielectric properties of the dielectric medium. The dielectric material is made out of atoms and molecules and, when placed between the plates of the charged-up capacitor, the negative charges in the dielectric are going to get attracted to the positive electrode of the capacitor. But those negatives cannot travel to the positive conductor, since this dielectric is a nonconducting material. However, the negatives can shift or lean towards the positive conductor.

This causes the charge in the atoms and molecules within the dielectric to become polarized. In the case of a soil medium saturated with water, it is also possible that the dielectric material started off polarized because some water molecules are naturally polarized. In this case, when the dielectric is placed between the charged-up conductors, the attraction between the negative side of the polarized molecule and the positive side of the capacitor would cause the polarized molecules to rotate, allowing the negatives to be a little bit closer to the positively charged conductor of the capacitor.

The general equation for calculating parallel plate capacitance is as follows:

(A3) $$C=\varepsilon \frac{A}{D}$$

where $\varepsilon =\varepsilon_{0}\varepsilon_{r}$ is the dielectric permittivity of the medium, obtained as the product of the dielectric permittivity of free space ($\varepsilon_{0}=$ 8.9 × 10^−12^ F/m) and the relative permittivity ($\varepsilon_{r}$), *A* is the area of each conductor, and *D* is the distance between them.

In the proposed monitoring system, the soil moisture module is designed with a parallel wire geometry, employing cylindrical conductors of the same diameter, each featuring a hollow core. In this case Equation (A2) can be rewritten as [60]:

(A4) $$C=\frac{\varepsilon \pi }{\cosh^{-1}\left(s/d\right)}$$

where *d* is the outer diameter of the electrode and *s* is the distance between them (from center to center).

With reference to this specific geometry, we may observe that the capacitance is affected by the relative permittivity, $\varepsilon_{r}$, of the region which is external to both conductors (i.e., the space surrounding the electrodes) if the internal volume is filled with air or adequately insulated. Figure A1 shows three different zones: (i) R1 (inside the conductors) where, as stated above, $\varepsilon_{r}$ of the conductor itself and that of the interior region does not impact *C*, (ii) R2 (grey-shaded region) where $\varepsilon_{r}$ impacts *C* to some degree, and (iii) R3 (between conductors, yellow region), where $\varepsilon_{r}$ has the largest impact on *C*.

## Appendix B. Supplementary Information

This section provides supplementary information essential for constructing the proposed monitoring system. All the essential files (including PCB fabrication files, sensor firmware file, 3D printing files, etc.) for building the monitoring system are available as Supplementary Material accompanying this paper. The Supplementary Material folder is organized as follows:

1. Board Fabrication Files folder:
  (a) The Gerber files (SHYPROM.kicad_pcb.zip);
  (b) The Centroid file (Centroid_SHYPROM.kicad_pcb.xls);
  (c) The Bill of Materials (BoM: SHYPROM_BOM.xls; only electric components).

These files are crucial for PCB implementation in specialized electronics manufacturing services. All the electronic components can be acquired in different electronics stores (e.g., JLCPCB, Mouser, Digikey, RSOnline, etc.). The purchase links to electric components can be found directly in the BoM spreadsheet.

2. SHYPROM Firmware folder:
  (a) Source code developed in Arduino IDE used to program the proposed monitoring system (SHYPROM.ino);
  (b) Source code for the SHYPROM_APP (only for Android-based operating systems devices).
3. 3D Printing Materials folder:
  (a) SHYPROM_BOX Folder: external box (waterproof), .stl files for containing the monitoring system;
  (b) SHYPROM_RINGS Folder: .stl files for quick assembling capacitive electrodes on the plexiglass tensiometers;
  (c) How to assemble SHYPROM Sensor: Quick tutorial useful for realizing and assembling the SHYPROM monitoring system.

The 3D printing models were developed in Fusion 360 software, version 2.0.18961 (https://www.autodesk.com).

For implementing the electronic part of the SHYPROM monitoring system, two options are possible. The first option is to construct the board by yourself, following the electrical schematic in Figure 2. The estimated cost of fabricating one piece, including materials, is approximately USD 15–20 for the ESP32SIM800L board, USD 50–55 for two MPX5100DP pressure transducers, and around USD 2 for the other required components (listed in the BoM file as a reference).

For field applications, it is advisable to utilize a specialized electronics manufacturing service capable of producing low-cost printed circuit boards (PCBs). This option requires the Gerber and BoM files (refer to the Board Fabrication Files folder in the Supplementary Material). In this case, fabrication costs are around USD 30 for printing five PCBs, excluding shipping.

The SHYPROM monitoring system also requires materials for the two probes that serve as tensiometers and for installing the electrodes of the capacitive-based module. This part of the sensor can be constructed using (i) plexiglass tubes with an outer diameter of 20 mm and (ii) 3 cm long cylindrical tubes that act as electrodes (refer to local or online markets for sourcing these materials). To simplify the assembly of the capacitive module, consider 3D printing PLA cylindrical elements (see Probe Ring Bottom PLA.stl and Probe Ring PLA.stl files in the SHYPROM_RINGS folder). These PLA elements were designed for correctly wiring the electrical cables and securing the electrodes along the plexiglass tubes.

Once the SHYPROM monitoring system is assembled, the next step is to load the firmware file into the ESP32SIM800L control unit (refer to SHYPROM.ino file, available for download from the SHYPROM Firmware folder). Before loading the firmware, it is crucial to set your Cloud URL (row 30 of the SHYPROM.ino file) and your Write API Key value (row 34 of the SHYPROM.ino file); these parameters depend on the chosen Cloud platform. The Firmware SHYPROM.ino file can be managed using the Arduino IDE, version 2.3.2 (https://www.arduino.cc/en/software).

Finally, the SHYPROM available data can be also visualized on the SHYPROM Android app. To install the app on your device, you need the .apk file, which can be generated from the SHYPROM_APP.aia file (available for download from the SHYPROM Firmware folder). The latter file contains the source code and can be directly opened and modified on the MIT APP INVENTOR online IDE (https://appinventor.mit.edu/). The code must be rearranged for your specific requirements (i.e., it is necessary to modify the requested home URLs according to your Cloud specifications). Once modified, the MIT APP INVENTOR IDE creates the .apk file, which is the source file required for installation on Android-based mobile devices.
