# Supplementary material for: Development and Application of an IoT-Based System for Soil Water Status Monitoring in a Soil Profile
Source: Sensors (Basel). 2024 Apr 25;24(9):2725. doi: 10.3390/s24092725 (PMC11086235; doi:10.3390/s24092725)

Quick guide

This brief guide presents the 3D-printed components necessary for assembling the SHYPROM monitoring system. Additionally, it provides details on the sensor's construction process, particularly focusing on assembling the capacitive-based module onto the plexiglass tubes, which, as mentioned in the paper, also serve as tensiometers.

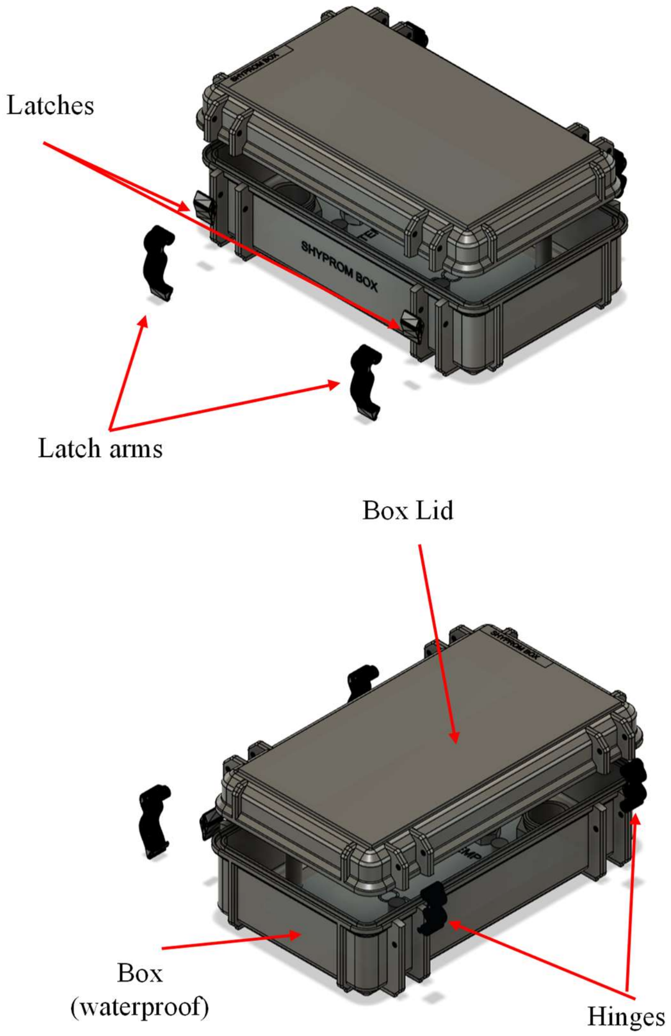

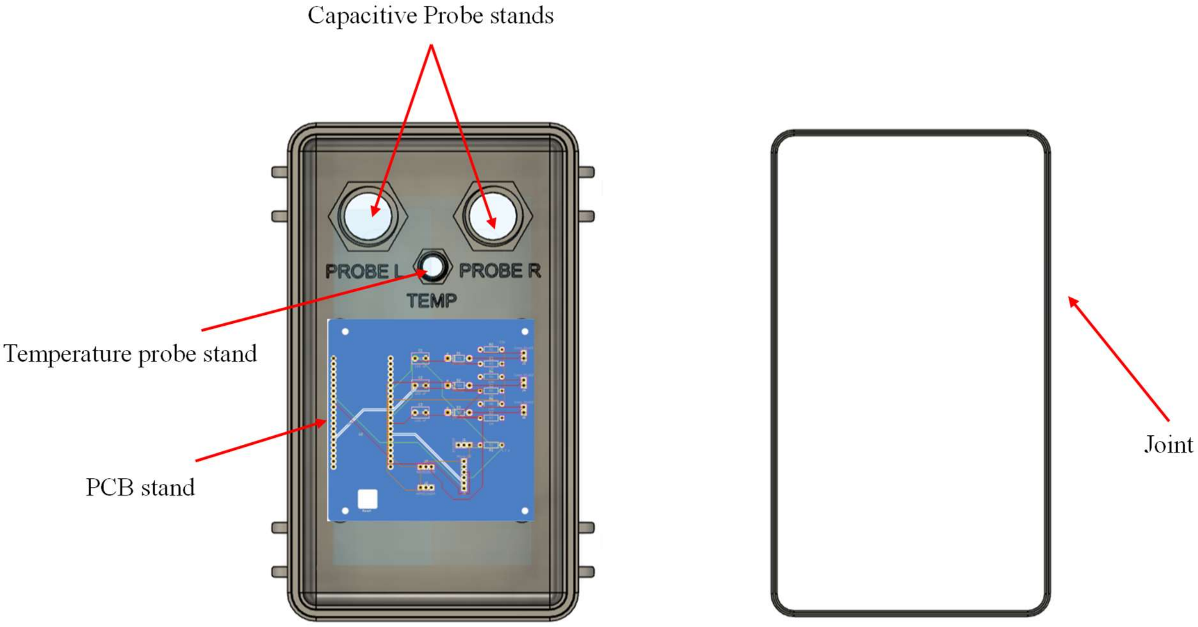

How to realize the capacitive unit

The SHYPROM monitoring system, as known, has the possibility to measure volumetric water content at different depths. At each depth, these measurements are possible when a couple of electrodes are placed in the soil profile. The electrodes have a tubular shape and are made of stainless steel 304. These cylinders are 3 cm high and 2 mm thin and were positioned on the plexiglass-tensiometers tubes, forming pairs of electrodes. To prevent sensor deterioration, after installation, the electrodes can isolated by spraying with a (water-based) paint.

Each electrode is soldered with a wire and the wire is connected to the PCB (see figure 2a and b of the paper). Connections are located in the top right of the PCB. The wire must be soldered in the internal part of the metallic cylinder (i.e., the electrode). Once soldered the wire, the electrode may be finalized using a couple of 3D PLA (see figure below) the metallic cylinder, and the two PLA components must be located on the plexiglass tube and then glued with a silicone paste to prevent electrode deterioration.

In summary:

Electrode Construction: The electrodes are cylindrical and made of stainless steel 304. Each electrode is 3 cm high and 2 mm thin.

Positioning: These electrodes are positioned on plexiglass-tensiometer tubes to form pairs at different depths within the soil profile.

Isolation: To prevent sensor deterioration, the electrodes are isolated after installation by spraying them with a water-based paint.

Wiring: Each electrode is soldered with a wire, and these wires are connected to the PCB (Printed Circuit Board), with the connections located in the top left of the PCB.

Soldering: The wire must be soldered to the internal part of the metallic cylinder (electrode).

Finalization: Once soldered, the electrode is finalized by enclosing it with a couple of 3D PLA components. These components are placed on the plexiglass tube and glued with silicone paste to prevent electrode deterioration.

Example of electrode assembling

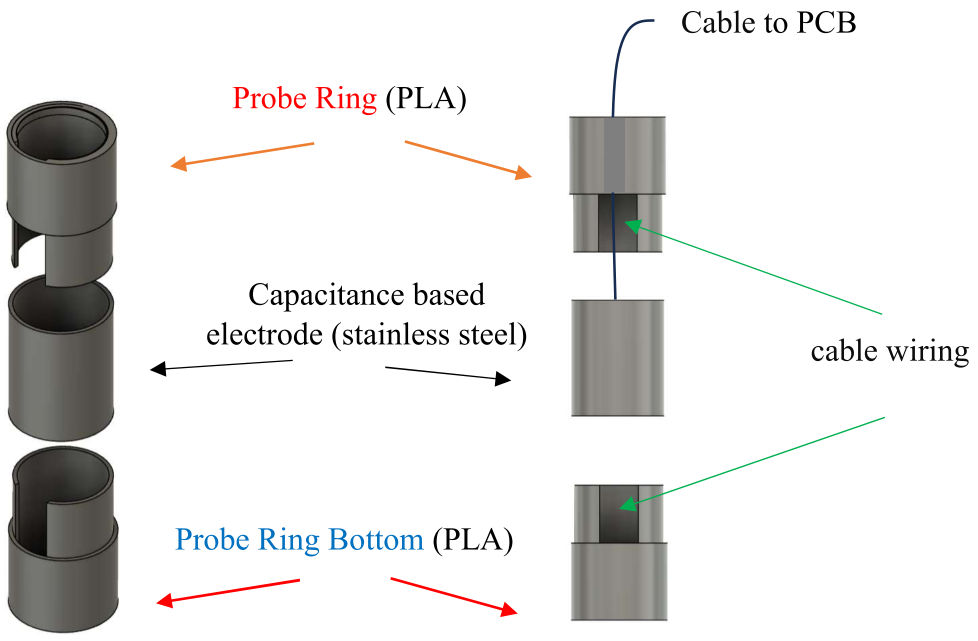

Supplement: Supplementary file 1 [file sensors-24-02725-s001.zip › sensors-2929232-supplementary/Supplentary Material/03 3D Printing Materials/How to assemble SHYPROM Sensor.pdf]
